# Crystal structure of DnaT^84–153^-dT10 ssDNA complex reveals a novel single-stranded DNA binding mode

**DOI:** 10.1093/nar/gku633

**Published:** 2014-07-22

**Authors:** Zheng Liu, Peng Chen, Xuejuan Wang, Gang Cai, Liwen Niu, Maikun Teng, Xu Li

**Affiliations:** 1 Hefei National Laboratory for Physical Sciences at Microscale and School of Life Sciences, University of Science and Technology of China, Hefei, Anhui 230026, People's Republic of China; 2 Key Laboratory of Structural Biology, Chinese Academy of Sciences, Hefei, Anhui 230026, People's Republic of China

## Abstract

DnaT is a primosomal protein that is required for the stalled replication fork restart in *Escherichia coli*. As an adapter, DnaT mediates the PriA-PriB-ssDNA ternary complex and the DnaB/C complex. However, the fundamental function of DnaT during PriA-dependent primosome assembly is still a black box. Here, we report the 2.83 Å DnaT^84–153^-dT10 ssDNA complex structure, which reveals a novel three-helix bundle single-stranded DNA binding mode. Based on binding assays and negative-staining electron microscopy results, we found that DnaT can bind to phiX 174 ssDNA to form nucleoprotein filaments for the first time, which indicates that DnaT might function as a scaffold protein during the PriA-dependent primosome assembly. In combination with biochemical analysis, we propose a cooperative mechanism for the binding of DnaT to ssDNA and a possible model for the assembly of PriA-PriB-ssDNA-DnaT complex that sheds light on the function of DnaT during the primosome assembly and stalled replication fork restart. This report presents the first structure of the DnaT C-terminal complex with ssDNA and a novel model that explains the interactions between the three-helix bundle and ssDNA.

## INTRODUCTION

A faithful and reliable transmission of genetic material requires precise coordination and regulation of chromosome replication (1,2). This process requires the assembly of a replication complex, a primosome, at the origin (3). A primosome consists of several proteins, including helicase, primase and several auxiliary proteins that are responsible for creating RNA primers on ssDNA during DNA replication (3,4). The assembly of the primosome is a fundamental step for both normal chromosomal replication and the stalled replication fork restart (5–7).

There are two types of primosomes for *Escherichia coli* chromosomal replication (3,8). The first type is the high fidelity DnaA-dependent *ori*C primosome, which uses DnaA as the initiator protein to bind to the *ori*C site and starts the assembly of the enzymatic replisome machine (9,10). Briefly, DnaA recognition and binding to *ori*C recruits DnaB helicase with the help of the helicase loader DnaC and coordinates the DnaB helicase and the primase DnaG functions (1,11–14). The second primosome is the *ori*C-independent PriA primosome, which requires PriA to recognise the n′-*pas* (primosome assembly site) and initiate the primosome assembly (5,8,15). In general, the PriA binds to n′-*pas* while assisted by the auxiliary PriB, PriC, DnaT, to recruit the DnaB, DnaC and DnaG (16). The two distinct primosomes share the same helicase DnaB and DnaG primase systems.

To the best of our knowledge, the model for the *E. coli* PriA-dependent primosome can be divided into the following steps (5): (i) PriA binding to the n′-*pas* induces PriB binding, to form a PriA-PriB-DNA complex (17,18). (ii) DnaT is recruited to the PriA-PriB-DNA ternary complex, perhaps to recruit the DnaB/C complex (5,19). (iii) DnaB is loaded from the DnaB/C complex onto ssDNA (2,11). (iv) DnaG is recruited for RNA primer synthesis (14).

The effective ability to restart the stalled or collapsed replication forks and repair damaged DNA is crucial for cell survival upon encountering DNA damage-inducing agents or in a nutrition-insufficient environment (3). Although the process of reloading replisomes onto abandoned replication forks has been reported in both bacteria and eukaryotes, only the bacterial factors, the PriA-dependent primosome, that mediate this process have been identified. Thus, the bacterial replication restart proteins serve as models for understanding the general mechanisms of replication restart in all cells (15). Until now, the structural and functional studies of PriA, PriB, DnaB, DnaC and DnaG have provided a substantial amount of information on the PriA-dependent primosome (2,13–15,20–23). Some research progress about DnaT has also been reported. The *E. coli* DnaT protein is originally discovered to be a critical factor for *E. coli* pBRR322 plasmid DNA replication and phiX174-type primosome assembly and is considered to be protein i (19,24). Genetic analysis of *E. coli* DnaT suggests that it is an essential replication protein for bacterial cell growth because the *dnaT822* mutant shows involvement with colony size, cell morphology, inability to properly partition nucleoids, ultraviolet (UV) sensitivity and basal SOS expression, which is similar to in *priA2::kan* mutants (25). Increasing the amount of DnaT in a reaction can circumvent the need for PriB in that reaction and can rescue the defects of certain mutant PriA proteins (19). A recent article reported that DnaT is also essential for *E. coli* growth at high hydrostatic pressure (26). In summary, the ssDNA binding protein DnaT, the N-terminal end of which is crucial for PriB binding, exists as a trimer in solution (5,27–30). However, the mechanism of how DnaT is recruited to the PriA-PriB-DNA complex and how it binds to the exposed ssDNA is still unclear. Additionally, little is known about the fundamental role of DnaT in the recruitment of DnaB helicase. Consequently, the atomic resolution structure of DnaT is urgent for our understanding of its function in ssDNA binding and in protein interactions during the primosome assembly.

Here, we report the crystal structure of the DnaT^84–153^-dT10 ssDNA complex structure, which adopts a novel ssDNA binding motif using the three-helix bundle mode. Biochemical and structural information shows that the DnaT can cooperatively bind to ssDNA to form rod-like nucleoprotein filaments, which indicate that the DnaT-ssDNA complex might function as a scaffold during primosome assembly.

## MATERIALS AND METHODS

### Cloning, expression and purification

All of the DNA fragments of *E. coli* DnaT were amplified from strain K12 genomic DNA by a polymerase chain reaction (PCR) using PrimeSTAR HS DNA Polymerase (TaKaRa). The PCR fragments were digested by restriction endonucleases BamH I and Xho I and inserted into a modified pET-28a (+) (Novagen) with a His-MBP-TEV cleavage site tag in the N-terminus of the recombinant protein. All of the DnaT mutants were generated from the recombination *wt-*DnaT pET-28a vector by site-directed mutagenesis (TaKaRa MutanBEST Kit). The mutant proteins were purified using the same procedure as that for the wide-type protein. For protein expression, the plasmid was transformed into *E. coli* BL21 (DE3) (Merck). Cells were grown in Luria-Bertani medium that contained 50 μg/ml kanamycin at 37°C and were induced with 1 mM isopropyl-β-D-thiogalactoside at 16°C for 20 h when OD_600_ reached 0.8. The cells were harvested by centrifugation at 6000 *g* for 10 min and were disrupted by sonication in buffer N (50 mM Tris-HCl, pH 7.5, 400 mM NaCl and 5% v/v glycerol). The recombinant proteins were purified by Ni-chelating resin (GE Healthcare, Waukesha, WI, USA) in buffer N, and then, they were dialysed against buffer A (20 mM Tris-HCl, pH 7.5, 40 mM NaCl and 5% v/v glycerol), to remove the His-MBP tag by TEV digestion at 4°C overnight. The proteins were further purified using HisTrap HP (GE Healthcare), HiTrap DEAE FF (GE Healthcare) and HiLoad 16/60 Superdex 200 (GE Healthcare) separately. The final proteins were pooled to 8–12 mg/ml in buffer A and stored at −80°C.

To prepare the SeMet-derivative protein, DnaT protein was expressed in *E. coli* strain B834 (Novagen) using M9 medium supplemented with SeMet and six amino acids, including threonine, lysine, phenylalanine, valine, leucine and isoleucine. The SeMet-derivative DnaT protein was purified essentially as described above.

### Crystallisation

Before crystallisation, DnaT was concentrated to 2 mg/ml in buffer A. Both full-length DnaT and DnaT mixed with the single-stranded DNA (dT15) at a molar ratio of 1:1.2 yielded crystals, but only the latter one, which was grown in 0.1 M MES monohydrate, pH 6.3, 17% (w/v) polyethylene glycol 6000, diffracted well enough to allow data collection. Both the native and SeMet-derivative DnaT were incubated with ssDNA at 25°C for 30 min before crystals were grown by hanging drop vapour diffusion at 14°C. It is worthwhile noting that the crystals first appeared as fine needles in 3–4 days that were too small to diffract well (Supplementary Figure S1A). After ∼1–2 weeks, some small stick shapes in the crystals arose, and these were large enough to diffract well for data collection (Supplementary Figure S1B). However, those crystals cracked after 2–3 weeks (Supplementary Figure S1C). Finally, all of the crystals disappeared after ∼3–4 weeks (Supplementary Figure S1D).

### Data collection and structure determination

For the data collection, all of the crystals were transferred into a cryoprotectant solution that consisted of the respective reservoir solution supplemented with 30% (v/v) glycerol, and then, they were flash frozen in liquid nitrogen. All of the data were collected at beamline BL17U of Shanghai Synchrotron Radiation Facility (SSRF) and processed with the HKL2000 package and programs in the CCP4 suit (31,32). For the SeMet-derivative DnaT^84–153^-dT15 complex crystal, data were collected at the wavelength near the selenium absorption edge. The single wavelength anomalous scattering data of DnaT^84–153^-dT15 collected from a single crystal was sufficient to calculate the initial phase using PHENIX.AutoSol (33), and a figure of merit (FOM) of 0.44 and 0.66 was acquired before and after the density modification. Then, the phase information was used to build an initial model using PHENIX.AutoBuild (33). The initial model was then completed through several cycles of manual model building in COOT (34) and refinement in Refmac5 (35). The refined model was adopted as a search model for the native 2.83 Å DnaT^84–153^-dT10 complex crystal structure determination using Molrep (36) in the CCP4i suite (32). The 2.83 Å complex structure model was then completed through several cycles of manual model building in COOT (34) and refinement in Refmac5 (35) until all of the electronic density in the map could be interpreted well and all of the residues had acceptable chemical conformation with the refinement statistics listed in Table 1. Both of the structures were checked using Molprobity (37), and all of the figures were prepared using Pymol (DeLano Scientific).

**Table 1.** Data collection, phasing and refinement statistics

	Se-Met-DnaT^84–153^ -dT10^a^	Native-DnaT^84–153^-dT10
**Data collection**		
Wavelength(Å)	0.97917(peak)	1.54001
Space group	P 1	P 1
Cell dimensions		
*a, b, c* (Å)	47.03, 46.96, 54.70	46.41, 46.69, 54.39
*α, β, γ* (°)	88.04, 86.79, 70.22	87.33, 86.01, 70.20
Resolution (Å)	34.62 - 2.08	38.02 - 2.83
	(2.15 -2.08) ^b^	(2.93 - 2.83)
Unique reflections	25125 (2207)	9917 (951)
Rmerge (%)	0.081(0.335)	0.107(0.405)
I/σI	9.4(3.4)	13.6(4.6)
Completeness (%)	95.6/95.2	96.9(96.5)
Redundancy	3.7	3.7
**Phasing**		
No. of sites (found/all)	8/10	
FOM	0.44	
FOM after RESOLVE	0.66	
**Refinement**		
Rwork / Rfree		0.184/0.238
Total No. atoms		3084
Residues		364
dT10		1
Waters		0
Mean B-factors		72.24
**R.m.s deviations**		
Bond lengths (Å)		0.0082
Bond angles (°)		1.1371
**Ramachandran plot (% residues)**		
Most favored		98.0
Additional allowed		2
Disallowed regions		0

^a^dT10 is short for the dT10 ssDNA.

^b^Values in parentheses are for highest-resolution shell.

### Fluorescence polarisation assays (FPAs)

FPAs were performed in buffer A at 20°C using a SpectraMax M5 microplate reader system. The wavelengths of fluorescence excitation and emission were 485 and 538 nm, respectively. Each well of a 384-well plate contained 200 nM fluorescent-labelled (5′-FAM) DNA probe and different amounts of *wt*-DnaT or mutants, with a final volume of 80 μl. For each assay, DNA-free controls were included. The sequences of substrates that were used in this study can be found in Supplementary Table S1. The fluorescence polarisation P (in mP units) was calculated using the equation P = (I_i_-I_)/(I_i_+I_). The fluorescence polarisation change P (in mP units) was fit to the equation ΔP = ΔPmax*[protein]^∧^*h*/(*K*_d_^∧^*h*+[protein]^∧^*h*) for one site-specific binding with the Hill slope, where *h* is the Hill coefficient. All of the experiments were performed three times, and the standard deviations of three repeated experiments were used to estimate the error.

### Polyacrylamide gel electrophoresis mobility shift assays

The interactions of purified recombinant proteins with different ssDNA substrates were assessed by polyacrylamide gel electrophoresis mobility shift assays. DNA-binding reactions (20 μl) were performed for 30 min at 20°C in buffer A, with the indicated protein concentrations and 6 μM dT30 or 20 μM dT10 DNA substrates. After the addition of 6 μl gel-loading buffer (50% glycerol, 0.02% bromophenol blue), the reaction mixtures were analysed by electrophoresis in 8% native polyacrylamide gels using 0.5×TBE buffer (45 mM Tris-borate, 1 mM ethylenediaminetetraacetic acid (EDTA), pH 8.0) at 4°C for 120 min and visualised by Gel-Red-staining.

### Agarose gel electrophoresis mobility shift assays

The interactions of purified *wt*-DnaT and its mutants with phiX174 ssDNA (Biolab) were assessed by agarose gel electrophoresis mobility shift assays. DNA-binding reactions (10 μl) were conducted for 30 min at 20°C in buffer A with the indicated protein concentrations and 50 nM phiX174 ssDNA substrates. After the addition of 2 μl gel-loading buffer (40% sucrose, 0.25% bromophenol blue), the reaction mixtures were analysed by electrophoresis in 0.8% agarose gels using 0.5×TBE buffer (45 mM Tris-borate, 1 mM EDTA, pH 8.0) at 4°C and visualised by Gel-Red-staining.

### Electron microscopy

Electron microscopy was used to examine the DnaT-phiX174 ssDNA complex with a published method that was applied for the analysis of the (39) PriB-phiX174 ssDNA complex (21). For preparing the DnaT-phiX174 ssDNA nucleoprotein filaments, the purified DnaT protein (8.0 μL; 10 mg/ml) was incubated with phiX174 ssDNA (1.6 μL; 50 μg/ml) and added buffer A to a final volume of 50 μl for 30 min at 20°C. The reaction products were diluted 4-fold with buffer A for Electron microscopy (EM) study. The final concentration of DnaT applied in the EM study was around 20 μM (400 μg/ml) and the substrate, circular phiX-174 ssDNA (5386 nt), was 0.24 nM (0.4 μg/ml) which was quite low. Other samples were prepared similar to this. For each sample, ∼3 μl protein-ssDNA complex was applied to a glow-discharged carbon-coated 400-mesh Cu EM specimen grid. Then, the sample was stained by 0.7% (*w/w*) uranyl formate. We imaged the protein-ssDNA particles under a low-dose condition using an FEI Tecnai F20 microscope (200 kV accelerating voltage, ∼0.6–0.8 μm underfocus) with an FEI Eagle CCD camera at 62 000× or 150 000× magnification. The final pixel sizes were 3.54 or 1.46 Å after 2-fold pixel averaging.

### Circular dichroism spectroscopy

The Circular dichroism (CD) spectra of the purified *wt*-DnaT and its mutants (0.1 mg/ml) were recorded at 20°C on a Jasco-810 spectropolarimeter at wavelengths from 190 to 260 nm. All of the samples in 40 mM sodium phosphate buffer (pH 7.5) were loaded into a quartz cuvette (*d* = 0.1 cm path length). A buffer-only sample was used as a reference. All of the CD spectra represent the average of three successive spectra. The molar ellipticities (*θ*) were plotted versus the wavelength, and the reference curve was subtracted from each curve. The percentages of the secondary structures were estimated using the online program *CAPITO* (38).

### Size-exclusion chromatography assays

Size-exclusion chromatography assays were performed on a Superdex 75 column (10/300 GL) (GE Healthcare) or a Superdex 200 column (10/300 GL) (GE Healthcare). The molecular mass standards and all of the recombinant proteins were loaded onto the column and equilibrated in buffer A. The standard proteins (Sigma) were Aprotinin (6.5 kDa), Cytochrome (12.4 kDa), Carbonic Anhydrase (29 kDa), Albumin (66 kDa), Alcohol Dehydrogenase from yeast (150 kDa) and Blue Dextran (2000 kDa).

## RESULTS

### Overall structure of the DnaT^84–153^–dT10 complex

To obtain insight into the DnaT function during primosome assembly, we performed structural research on the DnaT and its interactions with ssDNA. Although we obtained many types of DnaT crystals, no diffracted crystal was obtained. Next, we attempted to grow crystals by mixing DnaT with ssDNA (see Materials and Methods). We found that the crystal shapes changed from small needles to stick shapes during the growth. Then, 3–4 weeks later, all of the crystals disappeared and left brown precipitate (Supplementary Figure S1). We used sodium dodecyl sulphate-polyacrylamide gel electrophoresis to analyse the brown precipitate droplets or crystal droplets and found that the full-length DnaT that was used for crystal growth degraded into a 7 kDa fragment and the sequence range from Phe^89^ to Lys^143^ was confirmed by mass spectrometric analysis (Supplementary Figure S2).

**Figure 1.**
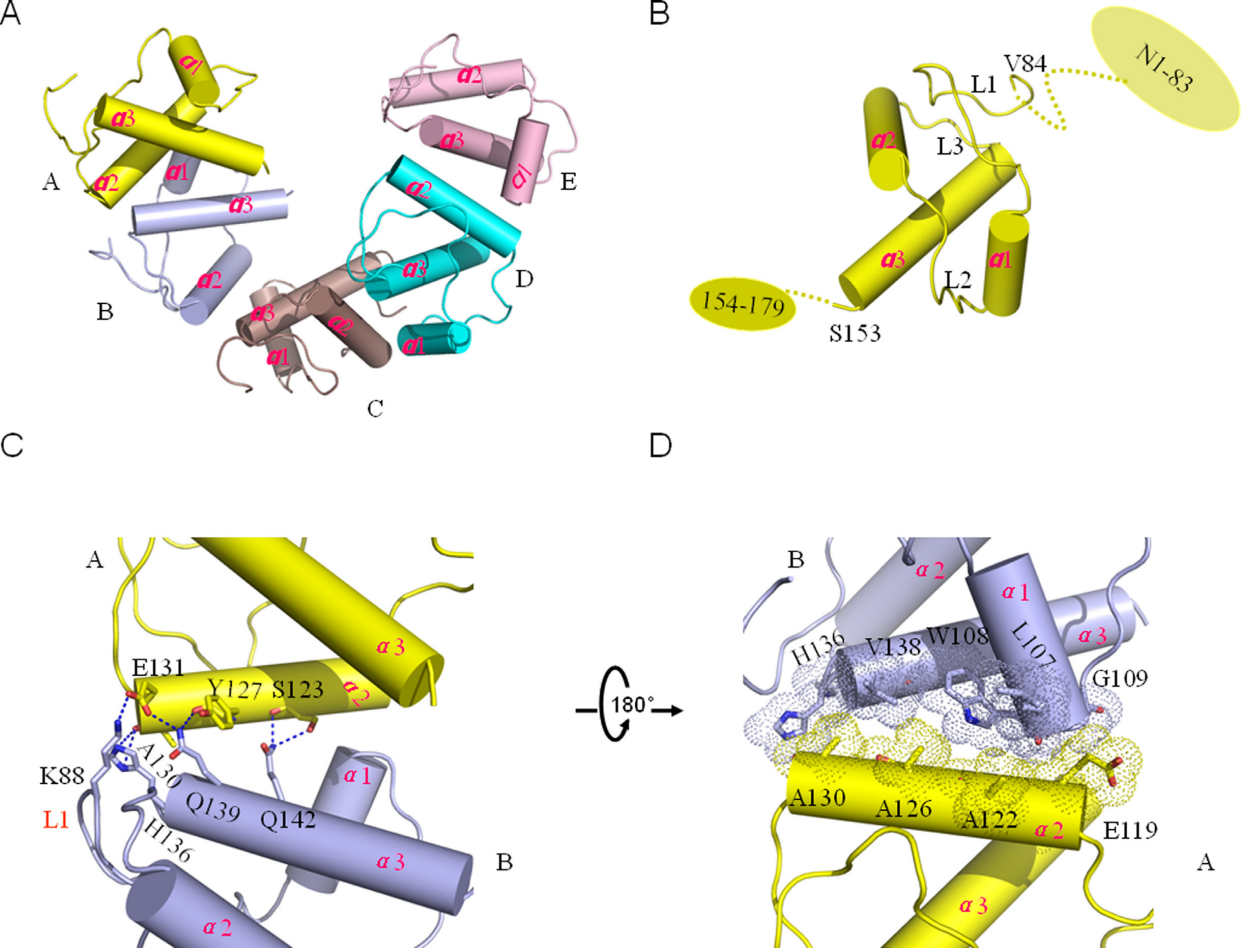
Overall structure of the 2.83 Å DnaT^84–153^–dT10 complex structure and the dimeric interface. (A) Crystal structure of the DnaT^84–153^, with all five subunits labelled in different colours. The ssDNA was removed from the complex structure. (B) The secondary structure composition of the protomer. The dashed line represents the unknown regions. (C–D) The dimeric interaction interface between neighbouring subunits is mediated mainly through hydrogen bonds and hydrophobic interactions. The colour used is consistent with panel A.

Using single wavelength anomalous scattering phasing and molecular replacement, we determined the 2.83 Å resolution crystal structure of the DnaT^84–153^–dT10 complex, with the crystallography statistics data in Table 1 (Figure 1). Although we also determined a higher resolution 1.96 Å structure, the electron density of dT10 ssDNA was very poor (Supplementary Figure S3, Supplementary Table S2). Consequently, we focused on the 2.83 Å DnaT^84–153^–dT10 complex structure in this study. Except for the dT10 ssDNA ligand, there are five molecules that display as a C-shaped helical assembly in one asymmetry unit (Figure 1A). The protomers are similar to one other, having a root mean square deviation for the Cα atoms of no more than 0.3 Å; each protomer contains a three-helix bundle that has the α3 helix flanked by the α1 and α2 helixes (Figure 1B). This structural motif is most similar to the N-terminal DUF1767 (pfam08585) domain of RMI1(PDB: 4CHT) (39) from a Dali server search (Supplementary Figure S4, Supplementary Table S3). However, the DUF domain is a eukaryotic domain of unknown function which is often found on the N-terminus. Meanwhile, the biggest difference between the DUF1767 domain of RMI1 and DnaT^84–153^ three-helix bundle is an extra loop (Val^84^-Ala^99^) existed in the N-terminus of the DnaT^84–153^ three-helix bundle (Supplementary Figure S4). The extra loop should be essential for the stabilisation of the three-helix bundle from the structure analysis (data not shown) and McCool *et al.* also indicated that the *dnaT822*, an in-frame six-codon (87–92) deletion, shows colony size, cell morphology, inability to properly partition nucleoids, UV sensitivity and basal SOS expression similar to *priA2::kan* mutants (25).

The five molecules are mediated through dimeric interfaces between each two adjacent subunits. Each dimeric interface has a buried surface area of ∼390 Å^2^ (as calculated by the PISA server). Taking the A and B interface as an example, the interaction interface is mediated mainly through the α2 helix in subunit A and the L1 loop and the α1 and α3 helixes in subunit B using hydrogen bonds and hydrophobic residues (Figure 1C and D). The side chains of Ser^123^ and Glu^131^ and the main chains of Ser^123^, Tyr^127^ and Ala^130^ of the α2 helix in subunit A contribute to interacting with the side chain of Lys^88^ in loop 1, and the side chains of His^136^, Gln^139^ and Gln^142^ of the α3 helix in subunit B interact through hydrogen bonds (Figure 1C). The hydrophobic parts of Leu^107^, Trp^108^ and Gly^109^ in the α1 helix and the His^136^, Val^138^ in the α3 helix of subunit B form hydrophobic interactions with the side chains of Ala^122^, Ala^126^ and Ala^130^ in the α2 helix of subunit A (Figure 1D).

**Figure 2.**
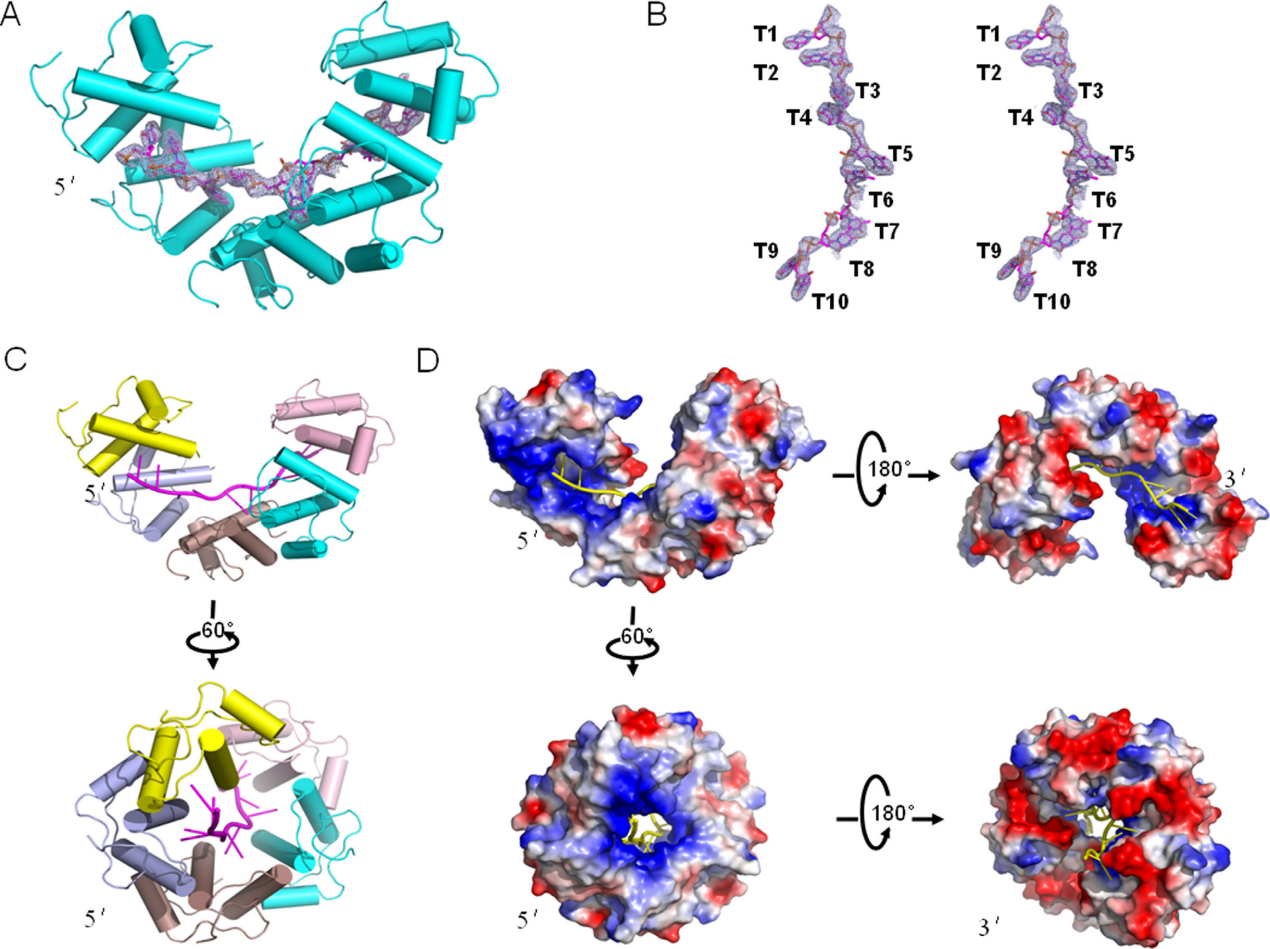
DnaT^84–153^-dT10 ssDNA complex structure. (A) The 2.83 Å DnaT^84–153^–dT10 complex structure with the 2Fo-Fc electron density contoured at 1.0-σ in the image. (B) The stereo view of the 2Fo-Fc electronic density. (C and D) The overall structure and electrostatic potential surface of the DnaT^84–153^-dT10 ssDNA complex from different views.

The ssDNA interaction cavity displays large positive charged regions and hydrophobic regions in the electrostatic potential surface (Figure 2A–C). The main positive charged regions distribute near the 5′-end of the ssDNA; however, the 3′-end appears to be mainly a negatively charged region (Figure 2D). The dimensions of the cavity can accommodate ssDNA only (not dsDNA) (Figure 2C and D). In the 2.83 Å resolution DnaT^84–153^-dT10 complex structure, five DnaT^84–153^ subunits bind to 10 nt of ssDNA, although we used the dT15 ssDNA for crystal growth (Figure 2A and B). We confirmed that DnaT can bind to 10 nt ssDNA using Electrophoretic mobility shift assay (EMSA) (Supplementary Figure S5A). Because the FAM (Carboxyfluorescein) in the short 10 nt 5′ labelled ssDNA might affect its binding to DnaT for FPAs, we also confirmed that DnaT can bind to dT15 or dA15 ssDNA via FPAs (Supplementary Figure S5B, Table 2).

**Table 2.** The ssDNA-binding parameters of wild type DnaT

wt-DnaT +ssDNA	Apparent *K*_d_ (μM)	*h*^a^
5F-dA15	46.7±6.87	2.5±0.6
5F-dT15	16.2±1.63	2.0±0.2
5F-dA30	39.2±3.85	1.8±0.2
5F-dT30	12.9±0.57	3.6±0.3
5F-dC30	25.7±1.74	2.8±0.4
5F-d(CA)_15_	31.5±3.60	2.3±0.3
5F-d(TG)_15_	1.30±0.11	1.6±0.2
5F-d(TGG)_10_	2.76±0.28	1.8±0.3
5F-d(TGGG)_7_	0.597±0.02	2.3±0.2

^a^Hill coefficient.

### Mode of the interaction of DnaT^84–153^ with ssDNA

**Figure 3.**
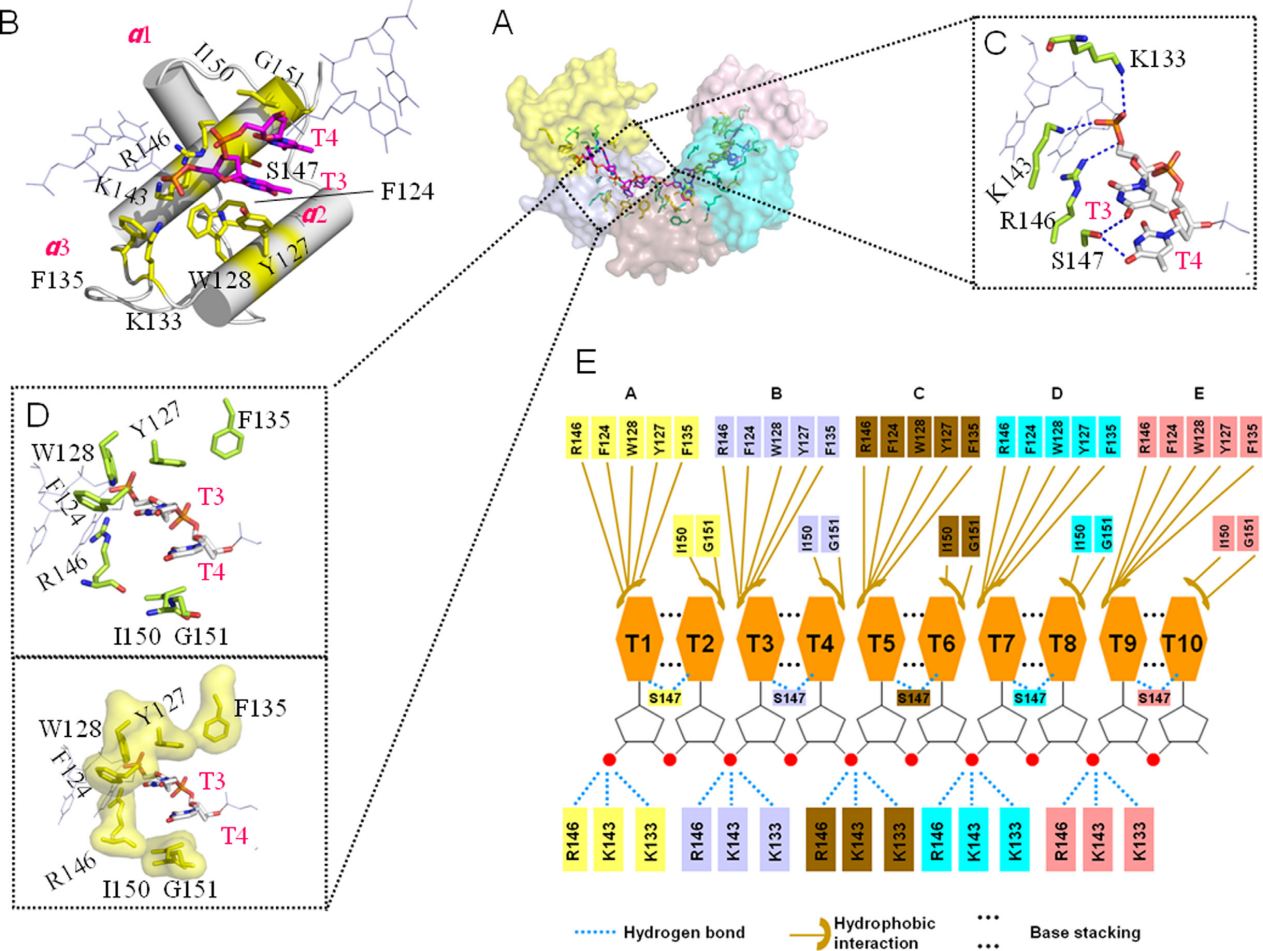
The interactions between DnaT^84–153^ and dT10 ssDNA. (A) The residues that are involved in ssDNA binding. (B) Each subunit (silver) can bind to 2 bases (magentas) using the α2 and α3 helixes and loop3. The involved residues are coloured yellow. (C) The hydrogen bond interactions. (D) The hydrophobic interactions are represented in the stick mode and the hydrophobic residues’ surface mode. (E) Schematic representation of the extensive interaction network. The colour of the amino acids is consistent with the surface colour in panel A.

In the 2.83 Å complex structure, almost all of the contacts between DnaT^84–153^ and dT10 are made through the DNA bases and phosphodiester backbone (Figure 3). The ssDNA binds in a hydrophobic and positively charged cavity at the junction between different subunits of the DnaT^84–153^, with each protomer binding to a 2-base unit using the α2 and α3 helixes (Figure 3A and B). The phosphodiester backbone is recognised mainly by Lys^133^, Lys^143^, and Arg^146^ through hydrogen bonds (Figure 3C), while the 2-thymine-base unit is stabilised through the hydrophobic centre formed by Phe^124^, Tyr^127^, Trp^128^, Phe^135^, Arg^146^, Ile^150^ and Gly^151^ (Figure 3D). Additionally, the Ser^147^ helps to stabilise the two bases through hydrogen bonds (Figure 3C). The Tyr^127^ and Ile^150^ stack against the two bases from each side of the two bases directly (Figure 3D). All of the interaction details of the DnaT^84–153^-dT10 complex are illustrated in Figure 3E.

**Figure 4.**
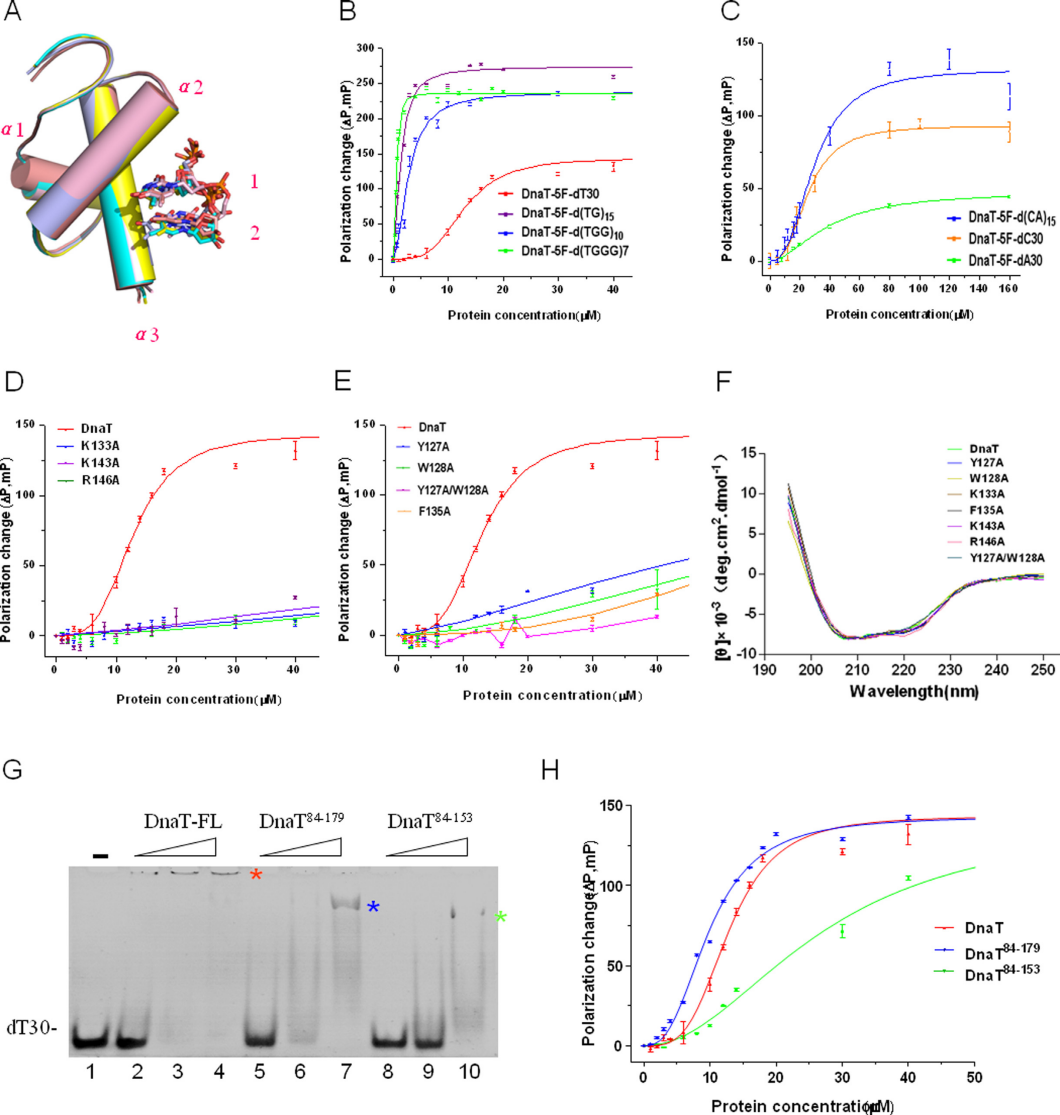
Alignment of double nucleotide segments and biochemical analysis of different versions of DnaT. (A) Alignment of double nucleotide segments. Structural superposition of protein chains A, B, C and D onto chain E with the corresponding two nucleotides of bound ssDNA differentially coloured to match the protein chains shown in Figure 1A. The double nucleotide numbering corresponds to even or odd numbers. (B and C) FPAs of *wt*-DnaT with different 5′-FAM-labelled ssDNA substrates. The sequences of the substrates that were used in this study can be found in Supplementary Table S1. The data on various ssDNA substrates were fitted according to the equation (see Materials and Methods). Error bars represent the standard deviation of three independent measurements. (D) FPAs of DnaT and the positively charged residue mutants with 5′-FAM-labelled dT30 ssDNA. (E) FPAs of DnaT and the aromatic residue mutants with 5′-FAM-labelled dT30 ssDNA. (F) Circular dichroism spectra of different DnaT variants. (G) EMSA result of DnaT-FL, DnaT^84–179^, DnaT^84–153^with dT30 ssDNA. 6 μM DNA substrate (dT30); proteins at lanes 1–4 (DnaT-FL): 0, 20, 60, 160 μM, respectively; lanes 5–7 (DnaT^84–179^): 20, 60, 160 μM, respectively; lanes 8–10 (DnaT^84–153^): 20, 60, 160 μM, respectively. The stars represent the position of protein-ssDNA complex bands. (H) FPAs of DnaT-FL, DnaT^84–179^, DnaT^84–153^ with 5′-FAM-labelled dT30 ssDNA. The data of DnaT-FL, DnaT^84–179^, DnaT^83–153^ were fitted according to the equation (see Materials and Methods). The error bars represent the standard deviations of three independent measurements.

Each base of the 2 bases stacks against the other, and all of the 5 DnaT^84–153^ subunits bind to the 2 bases in a similar way (Figure 4A). The first base of the 2-base unit can be recognised and stabilised by the large side chains of the 3 aromatic residues Phe^124^, Tyr^127^ and Trp^128^; specifically, however, the second one is recognised and stabilised only by the small Ile^150^ (Figure 3D). Consequently, the deviations of the odd number bases are smaller than the even number bases (Figure 4A). We conclude that this feature is fundamental to ensuring that DnaT binds to different types of ssDNA sequences.

To confirm this conclusion, we designed various ssDNA sequences (Supplementary Table S1) for the FPA experiments according to the following two rules. First, no hairpin structures were allowed. Second, there was no double-stranded DNA formation. We used the d(TGGG)_7_ to simulate 5′-FAM-labelled dG30 because the 5′-FAM labelling efficiency of dG30 chemically is usually very low. The FPA experiments indicated that DnaT could bind to dT30, dC30, dA30 and d(TGGG)_7_ cooperatively, with the Hill coefficients *h* and apparent *K*_d_ values listed in Table 2 (Figure 4B and C). For the purine and pyrimidine odd and even staggered arrangements d(TG)_15_, the apparent *K*_d_ is between the apparent *K*_d_ values of dT30 and d(TGGG)_7_; a similar consideration occurs with d(CA)_15_ (Figure 4B and C, Table 2). The apparent *K*_d_ *of* the purine and pyrimidine mix arranged in d(TGG)_10_ is also between the apparent *K*_d_ values of the dT30 and d(TGGG)_7_ probes (Figure 4B and C, Table 2). According to the FPAs data, we conclude that DnaT can bind to dA30 with lower affinity and DnaT can bind to d(TGGG)_7_ with higher affinity than all of the other tested sequences. These results demonstrated that there may be a certain preference for DnaT to bind to different types of ssDNA, but it has no sequence specificity in ssDNA binding, which is also consistent with the biological function of DnaT.

Consequently, the interaction mode of the DnaT^84–153^–dT10 complex and the FPAs results indicate that DnaT can bind to different types of ssDNA, which is fundamental for its physiological substrate bindings.

### Critical residues for ssDNA binding

To evaluate the residues that are involved in ssDNA binding, we studied the effect of various mutations on ssDNA binding using FPA. For the positively charged phosphodiester backbone binding residues, we constructed K133A, K143A and R146A mutants. All of the three mutants affected the ssDNA binding ability greatly (Figure 4D). For the hydrophobic aromatic residues, we obtained the Y127A, W128A, F135A and Y127A/W128A double mutants. The Y127A, W128A and F135A also showed low binding ability to dT30. However, the double mutants Y127A/W128A displayed almost no detectable binding activity (Figure 4E). These mutations did not affect the protein structures, as indicated by indistinguishable CD spectra (Figure 4F). They did not destroy the assembly of DnaT either, as assessed via size-exclusion chromatography assays (Supplementary Figure S5C). Consequently, those positively charged and aromatic residues were critical for the dT30 ssDNA binding.

### Both the N and C terminals are essential for the cooperative effect

Our FPA experiments showed that DnaT can bind to all of the assayed ssDNA cooperatively, with the Hill coefficients listed in Table 2. The Hill function indicated that different subunits of DnaT cooperate for the ssDNA binding because DnaT existed as a monomer-trimer-oligomer equilibrium in solution (29,30,40).

Because the 2.83 Å resolution complex structure displayed as a degraded DnaT, we then analysed whether the degraded DnaT could bind to ssDNA using EMSA and FPA. We used different lengths of DnaT fragments, including the full-length DnaT, the N-terminal-truncated DnaT^84–179^ and the N- and C-terminal-truncated DnaT^84–153^, to simulate the DnaT degradation. The EMSA results indicated that the three fragments had a degradative property in dT30 ssDNA binding (Figure 4G). The FPA data revealed that the apparent *K*_d_ deviations of the DnaT and N-terminal-truncated DnaT^84–179^ to dT30 were inconspicuous; however, the *h* reduced apparently from 3.6 ± 0.3 to 2.7 ± 0.2 (Figure 4H, Table 3). Furthermore, the apparent *K*_d_ of the N- and C-terminal-truncated DnaT^84–153^ to dT30 increased from 12.9 ± 0.57 μM to 27.1 ± 2.3 μM, and *h* decreased further to 2.1 ± 0.2 when compared with full-length DnaT (Figure 4H, Table 3). Additionally, the DnaT^84–179^ and DnaT^84–153^ fragments existed as dimers in solution, which was distinct from the DnaT trimer state also (Supplementary Figure S5D) (30). Therefore, the aggregation state changes would lead to a reduction in the cooperative effect on the ssDNA binding. The observations also revealed that both the N and C terminals were essential to having the cooperative effect.

**Table 3.** The ssDNA-binding parameters of different versions of DnaT with dT30

DnaT variant	dT30 Apparent *K*_d_ (μM)	*h*^a^
Wild-type	12.9±0.57	3.6±0.3
DnaT^84–179^	9.87±0.32	2.7±0.2
DnaT^84–153^	27.1±2.3	2.1±0.2

^a^Hill coefficient.

These results simulated the DnaT degradation that caused the trend in ssDNA release. Further degradations in the DnaT^84–153^ fragment might release the ssDNA ligand in the crystal and lead to crystal disappearance. Consequently, the incompleteness of the ligand electron density in the 1.96 Å resolution structure might be attributed to the protein degradation that caused ssDNA dissociation (Supplementary Figure S3).

### DnaT binds to phiX174 ssDNA and forms a nucleoprotein filament

**Figure 5.**
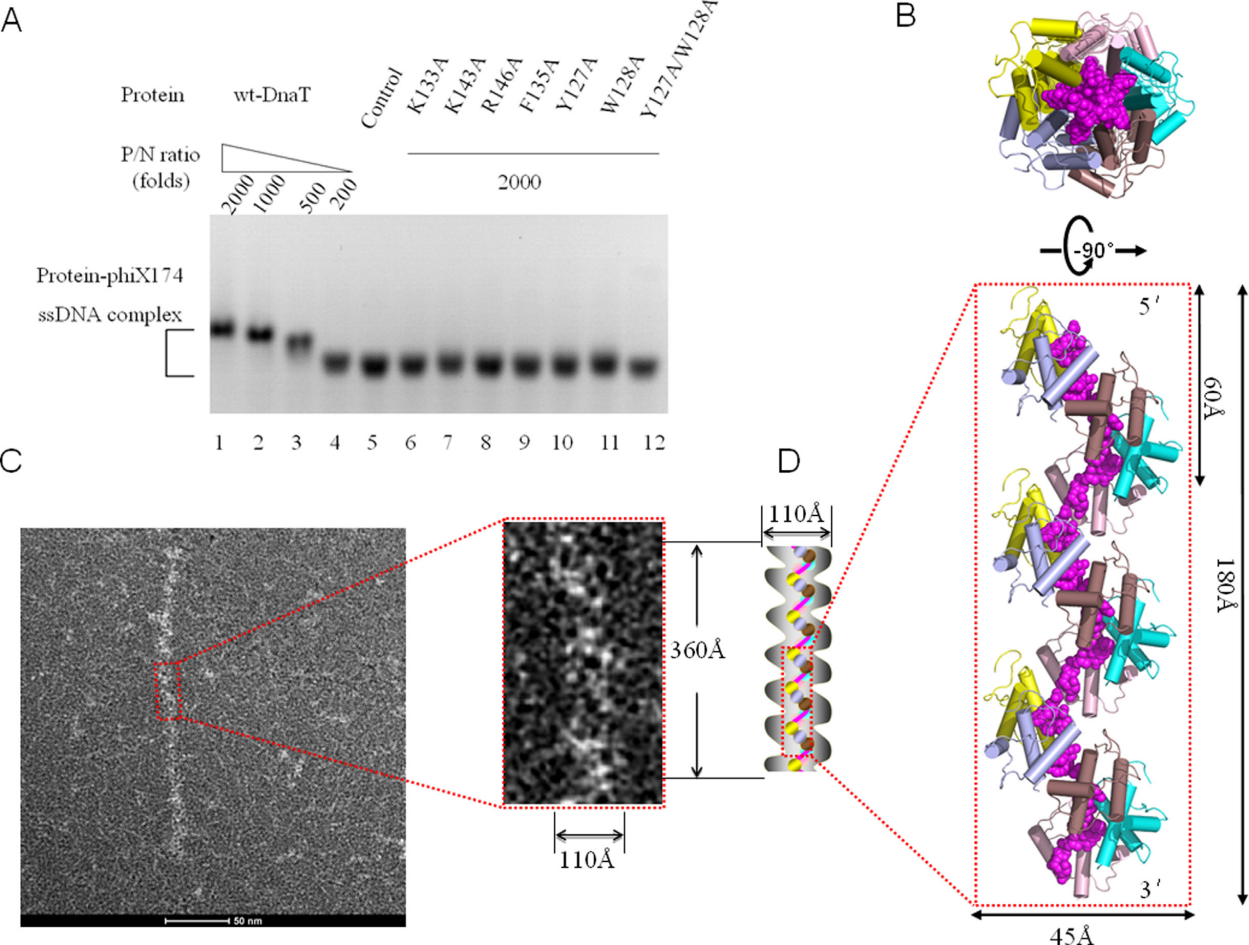
DnaT binds to phiX174 ssDNA to form rod-like nucleoprotein filaments. (A) Binding of wild-type or DnaT mutants to phiX174 ssDNA. The reaction solutions contained 50 nM circular phiX174 ssDNA and DnaT proteins at the indicated protein / nucleotide concentration ratio (P/N ratio). The protein concentrations used were 100 (lane 1), 50 (lane 2), 25 (lane 3), 10 (lane 4) and 0 μM (lane 5). The DnaT mutant protein concentration that was used was 100 μM (lanes 6–12). Bands correspond to unbound phiX174 ssDNA, and various DnaT-phiX174 ssDNA complexes were visualised by Gel-Red staining. (B) Side and top views of the oligomerised DnaT^84–153^–dT10 complex, reconstructed through crystal packing, showing 15 DnaT^84–153^ subunits and 3 strands of dT10 ssDNA. Colouring as in panel A in Figure 1. (C) DnaT-phiX174 ssDNA filament observed by negative staining EM. Enlarged view of the filament in the red box displays the width and length of the filament. (D) Model for the DnaT-phiX174 ssDNA filament. The grey jagged edge represents the possible relative position of the unknown structure of DnaT^1–83^ and DnaT^154–179^. The model width is set to 110 Å because the width of the model generated from the crystal structure is ∼45 Å, of which each protomer occupies 40% (in sequence length) of the full length DnaT. The model length is set to 360 Å because the helical pitch is 60 Å. At the same time, the N terminal (DnaT^1–83^) and short C terminal (DnaT^154–179^) might not affect the helical pitch as judged from the crystal structure model. The purple line represents ssDNA, while each ball (the colour of which is consistent with panel B) represents the protomer of the crystal structure.

To confirm whether the crystal structure of the DnaT^84–153^-dT10 complex and the results from the biochemical analysis of the protein with synthetic oligonucleotides are relevant to natural events, the binding of DnaT to circular phiX-174 ssDNA (5386nt) was analysed by agarose gel EMSA. By increasing the amount of DnaT that was added, a gradual decrease in the mobility of the phiX-174 ssDNA can be detected (Figure 5A, lanes 1–5). This observation probably reflects copies of DnaT that are bound to the phiX-174 ssDNA and slow down the mobility of the multicomponent complex. At the same time, the residues on DnaT that are crucial for dT30 binding are also important for phiX-174 ssDNA binding. All 7 mutants (Figure 5A, lanes 6–12) show a large effect on phiX-174 ssDNA binding. The mobility of the double mutant Y127A/W128A is almost identical to that of the control, which indicates that this version of mutant binds phiX-174 ssDNA poorly even at 2000 P/N ratios (Figure 5A, lane12).

In the DnaT^84–153^-dT10 complex, five DnaT^84–153^ molecules bind to one 10nt-ssDNA. Because it has been reported that both single-stranded DNA-binding proteins (SSBs) and PriB can act as sequence-independent ssDNA chaperones (41), which do not limit the conformation of the bound ssDNA to the extent that is observed for other known DNA-binding proteins (21), and because we did perform the experiments to identify the ability of DnaT binding to longer ssDNA (dT30), we modelled the longer helical filament shape oligomerised DnaT^84–153^ –ssDNA according to crystal packing (Figure 5B). The top and side views of the oligomerised DnaT^84–153^-ssDNA complex display as ring and helical filament shapes (Figure 5B). In this model, the DnaT^84–153^ molecules form a helical filament through dimer–dimer interactions with 5 DnaT^84–153^ proteins per turn (60 Å in helical pitch and 45 Å in diameter) (Figure 5B). To test this model, we performed the negative staining EM experiments. The control experiments indicated that the phiX-174 ssDNA is invisible under the experimental condition (Supplementary Figure S6A), however, the DnaT alone displays as the short rod-like filaments (Supplementary Figure S6B). This phenomenon reminded us whether the purified DnaT protein had already bound to the physiological substrates or the DnaT alone, without ssDNA, could also form filaments. In order to figure out the two possibilities, we added DNase I to the purified DnaT protein for EM analysis which shows a similar result as the DnaT alone (Supplementary Figure S6C). The two similar results illuminated that the possible physiological substrates of DnaT might be protected completely by DnaT or DnaT alone is also a filament. We then added short ssDNA or long ssDNA substrates to the purified DnaT for EM analysis. The results indicated that short ssDNA can shorten the possible filaments formed by DnaT itself, while long ssDNA will elongate them (Supplementary Figure S6D and E, S7A). The results helped to rule out the possibility that the purified DnaT had already bound to the physiological substrates. First of all, if the purified DnaT had already bound to the physiological substrates, it would be difficult for a lower affinity substrate dT50 ssDNA to compete with any physiological substrates. Secondly, our FPA results also indicated that DnaT might prefer to bind to substrates with random sequnces (Figure 4B and C, Table 2). Meanwhile, full length DnaT has also been reported to exist as monomer, trimer (30) or oligomer (40) depending on its different concentrations by several groups. Consequently, the DnaT alone might display as filaments also. This phenomenon can also be found in other ssDNA binding proteins, such as RecA which can display as filaments without adding ssDNA substrate (42).

The further EM experiments indicated that the DnaT^84–179^, phiX-174 ssDNA and DnaT^84–179^-phiX-174 ssDNA complex are invisible under the experimental condition (Supplementary Figure S7B and C). This phenomenon might be ascribed to two reasons. First of all, the DnaT^84–179^ itself exists as a dimmer in solution which is too small to be viewed by EM. Secondly, the dimeric DnaT^84–179^ might bind to phiX-174 ssDNA randomly (Supplementary Figure S7D and E). The model is also consistent with the different Hill coefficients to dT30 between full length and truncated DnaT^84–179^ version as the truncated version shows lower cooperative effect than full length DnaT (Figure 4H). It seems that the DnaT^84–179^ binding model conflicts with our crystal structure. Actually, it helps to understand why the crystal finally disappeared as we mentioned in the Materials and Methods.

Our EM results indicated that DnaT could form rod-like nucleoprotein filaments with phiX-174 ssDNA (Supplementary Figure S6E and F). To some extent, the dimensions of the proposed model are consistent with the morphology that is observed in negatively stained electron micrographs of DnaT-phiX174 complex (Figure 5B and C). Therefore, based on the EM results and the crystal structure, we proposed a new nucleoprotein filament model that can explain how DnaT binds to ssDNA and lay the foundation for future research on DnaT-related processes (Figure 5D).

## DISCUSSION

### DnaT^84–153^ -dT10 ssDNA complex revealed a novel ssDNA binding mode

**Figure 6.**
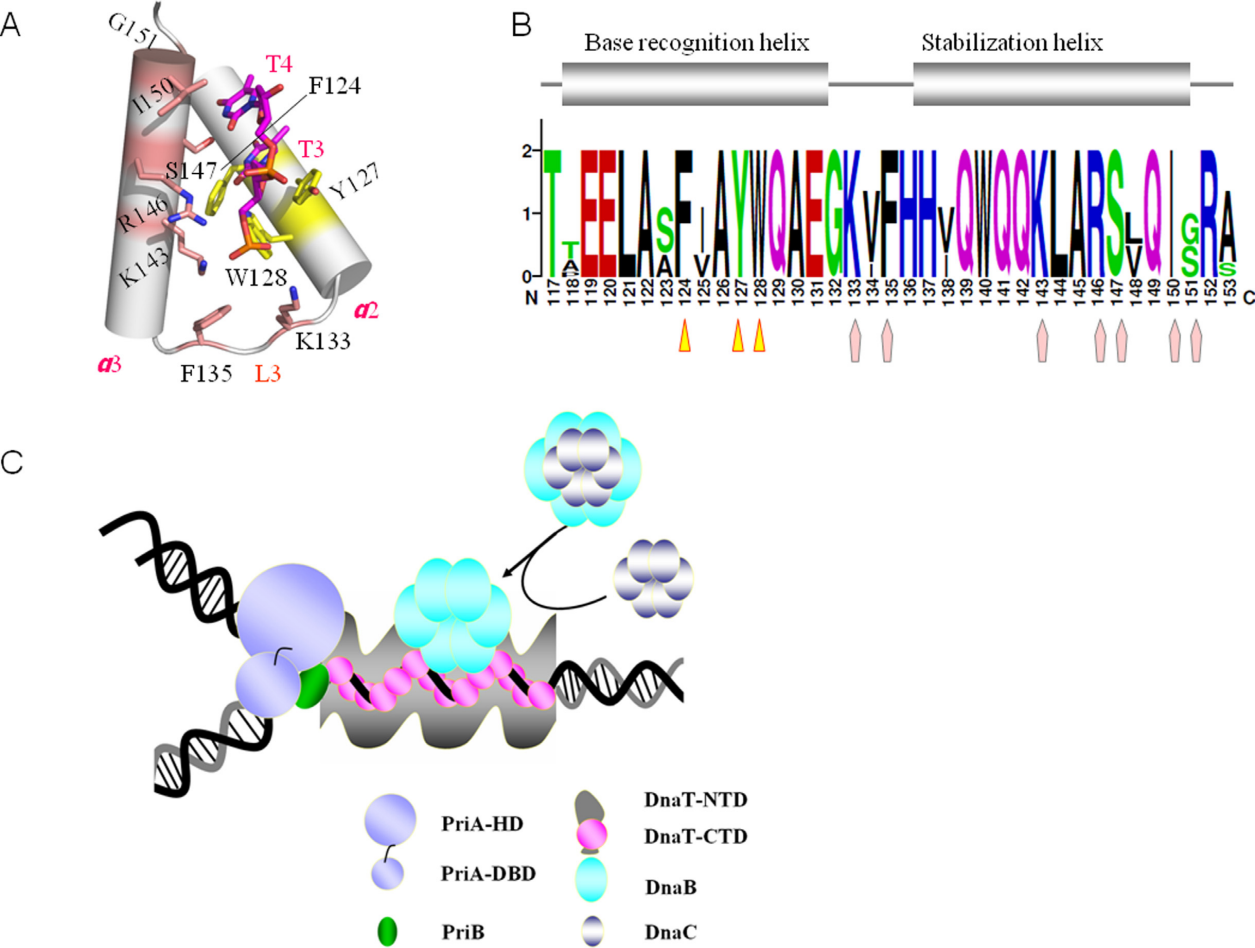
The ssDNA binding mode of DnaT^84–153^, an overview of the ssDNA-binding domains and a model for DnaT facilitating PriA-dependent primosome assembly. (A) The residues for base recognition are coloured yellow, while for base stabilisation, they are coloured brick red. (B) The sequence conservation of the base recognition helix (yellow triangle) and base stabilisation helix (brick red pentagon). (C) Model for DnaT facilitating PriA-dependent primosome assembly. Here, we sketched the outline of DnaT^1–83^ and DnaT^154–179^ in grey based on the result of the EM experiment, which revealed that DnaT could form nucleoprotein filaments with phiX174-ssDNA (Supplementary Figure S6).

Different types of ssDNA binding domains are evolved to adapt to multiple functions and fulfil their obligations during the life cycle. There are many structural topologies that have been well studied for ssDNA binding, such as oligonucleotide/oligosaccharide/oligopeptide-binding folds, K homology domains, RNA recognition motifs, RecA-like domain, whirly domain and a recently defined DrpA domain, among others (43,44). The structural and biochemical characterisation on *E. coli* DnaT that we reported here revealed a novel ssDNA-binding mode that is distinct from all of those mentioned. The *E. coli* DnaT displays a typical feature in recognising ssDNA using a base-inward fashion. DnaT^84–153^ recognises the bases of ssDNA mainly through the conserved Phe^124^, Tyr^127^ and Trp^128^ of the α2 helix, while the stabilisation of the phosphodiester backbone uses more residues, including Lys^133^, Phe^135^ in the L3 loop, and Lys^143^, Arg^146^, Ser^147^, Ile^150^ and Gly^151^ in the α3 helix (Figure 6A and B); it also combines multiple homologous domains that collaborate together to achieve the full activity level. However, both the fold and ssDNA binding mode of the DnaT^84–153^ three-helix bundle domain are distinct from those well-known ssDNA binding domains (43,44). The 2.83 Å DnaT^84–153^–dT10 complex structure showed a unique ssDNA binding mode that is different from any of the known RNA binding motifs (45). Consequently, this structure could be the first three-helix bundle that binds to the single-stranded DNA as far as we know.

### DnaT-ssDNA nucleoprotein filament might be a scaffold that is fundamental for the upcoming events

In this investigation, we determined the crystal structure of the DnaT^84–153^-dT10 ssDNA complex, which displays a spiral filament assembly that is adopted by many proteins that are involved in DNA replication, such as DnaA, RecA and PriB (21,46,47). However, the ssDNA binding mode is completely different because each subunit of DnaA or RecA can bind 3 bases, while each subunit of DnaT binds 2 bases. As the complex structure exhibited a degraded DnaT coiling around the ssDNA, we analysed different lengths of DnaT fragments, including the full-length DnaT, the N-terminal-truncated DnaT^84–179^ and the N- and C-terminal-truncated DnaT^84–153^, to simulate the DnaT degradation. The biochemical analysis results revealed that both the N- and C-terminals of DnaT are essential to having the cooperative effect. Combined with the binding assays we arrived at a conclusion that DnaT can bind to different types of ssDNA, which is fundamental for its physiological substrate bindings. The negative staining EM results also revealed that DnaT can bind to phiX-174 ssDNA and form the rod-like nucleoprotein filaments structure, which might illuminate a fundamental foundation of DnaT during the PriA-dependent primosome assembly and stalled replication fork restart. The complex structure of DnaT^84–153^-dT10 ssDNA and negative staining EM results that we report here provide us a good platform for further studies into DnaT and other related proteins.

A model emerges from our data to explain how DnaT functions during the primosome assembly for the replication restart (Figure 6C). As is known, the PriA-dependent primosome assembly is initiated by the binding of PriA to the C-terminal of SSB, to expose ssDNA from SSB, to which PriA can then bind (15). PriA binds the abandoned DNA replication forks and then exposes the PriB binding site and the additional ssDNA (5,17). PriB binds to the PriA helicase domain and to ssDNA either pre-existing at the replication fork or created by the helicase activity of PriA to form a PriA:PriB:ssDNA ternary complex (5). The functional experiments by Liu *et al.* indicated that the major role of PriB during primosome assembly is to facilitate formation of a complex containing both PriA and DnaT as high concentrations of DnaT could rescue the replication defects of the PriA mutants, and both wild-type and some mutant PriA proteins were shown to be able to form a complex with DnaT in the absence of PriB (19). Recruitment of DnaT to the PriA:PriB:ssDNA nucleoprotein complex can result in release of ssDNA by PriB (5). It was also reported that PriB could interact with DnaT directly through the N-terminal domain of DnaT (5,27). Combined with our novel EM results that full-length DnaT could bind to the ssDNA by forming a spiral filament, we propose that the interaction between DnaT and PriB would open the oligomeric DnaT, which further binds to the ssDNA cooperatively and stabilises the exposed ssDNA. Consequently, acting as a molecular wedge to strip ssDNA from the surface of PriB, DnaT undergoes a conformational change, switching an oligomeric form to a helical filament along the exposed ssDNA via its C-terminal domain, and at the same time, it interacts with PriB through the DnaT N-terminal domain, which causes a release of ssDNA by PriB. Then, the DnaB/C complex is recruited to the primosome, probably via direct contact between DnaB and the DnaT-ssDNA filament, which would occur simultaneously with DnaC dissociation (5). Nevertheless, the mechanism whereby the DnaB/C complex is targeted to the primosome is still unknown, more studies are required to determine the detailed interaction relationships that are involved in the mechanism by which DnaB is recruited by DnaT. Compared with the high fidelity DnaA-dependent *ori*C primosome, which uses DnaA as the initiator protein to bind to the *ori*C site and starts the assembly of the enzymatic replisome machine (9,10), PriA-dependent primosome chooses to assign those functions to three interactional proteins: PriA, PriB, DnaT as we described. For DNA damage repair, this might be an assembly level adjustment mechanism to accomplish the precise regulation.

Overall, our model provides insight into the molecular mechanisms that adapt the initial step of the PriA-PriB interaction and the DnaB/C recruitment of replication restart in bacteria. Through a combination of the DnaT cooperative ssDNA binding property and spiral filament arrangement, we speculate that DnaT can bind and stabilise the exposed ssDNA, which is useful for recruiting associated factors such as the DnaB/C complex for restarting a stalled replication fork during PriA-dependent primosome assembly.

## ACCESSION NUMBERS

The atomic coordinates and structure factors have been deposited in the PDB under accession code 4OU7 for the 2.83 Å DnaT^84–153^-dT10 ssDNA complex structure and 4OU6 for the 1.96 Å DnaT^84–153^-dT10 ssDNA complex structure.

## SUPPLEMENTARY DATA

Supplementary Data are available at NAR Online.

SUPPLEMENTARY DATA

## References

[1] Mott M.L., Berger J.M. (2007). DNA replication initiation: mechanisms and regulation in bacteria. Nat. Rev. Microbiol..

[2] Arias-Palomo E., O'Shea V.L., Hood I.V., Berger J.M. (2013). The bacterial DnaC helicase loader is a DnaB ring breaker. Cell.

[3] Masai H., Arai K. (1996). DnaA- and PriA-dependent primosomes: two distinct replication complexes for replication of Escherichia coli chromosome. Front Biosci..

[4] Allen G.C., Kornberg A. (1993). Assembly of the primosome of DNA replication in Escherichia coli. J. Biol. Chem..

[5] Lopper M., Boonsombat R., Sandler S.J., Keck J.L. (2007). A hand-off mechanism for primosome assembly in replication restart. Mol. Cell.

[6] Heller R.C., Marians K.J. (2006). Replication fork reactivation downstream of a blocked nascent leading strand. Nature.

[7] Gabbai C.B., Marians K.J. (2010). Recruitment to stalled replication forks of the PriA DNA helicase and replisome-loading activities is essential for survival. DNA Repair (Amst).

[8] Heller R.C., Marians K.J. (2005). The disposition of nascent strands at stalled replication forks dictates the pathway of replisome loading during restart. Mol. Cell.

[9] Erzberger J.P., Pirruccello M.M., Berger J.M. (2002). The structure of bacterial DnaA: implications for general mechanisms underlying DNA replication initiation. EMBO J..

[10] Liu B., Eliason W.K., Steitz T.A. (2013). Structure of a helicase-helicase loader complex reveals insights into the mechanism of bacterial primosome assembly. Nat. Commun..

[11] Makowska-Grzyska M., Kaguni J.M. (2010). Primase directs the release of DnaC from DnaB. Mol. Cell.

[12] Lo Y.H., Tsai K.L., Sun Y.J., Chen W.T., Huang C.Y., Hsiao C.D. (2009). The crystal structure of a replicative hexameric helicase DnaC and its complex with single-stranded DNA. Nucleic Acids Res..

[13] Naue N., Beerbaum M., Bogutzki A., Schmieder P., Curth U. (2013). The helicase-binding domain of Escherichia coli DnaG primase interacts with the highly conserved C-terminal region of single-stranded DNA-binding protein. Nucleic Acids Res..

[14] Bailey S., Eliason W.K., Steitz T.A. (2007). Structure of hexameric DnaB helicase and its complex with a domain of DnaG primase. Science.

[15] Bhattacharyya B., George N.P., Thurmes T.M., Zhou R., Jani N., Wessel S.R., Sandler S.J., Ha T., Keck J.L. (2014). Structural mechanisms of PriA-mediated DNA replication restart. Proc. Natl. Acad. Sci. U.S.A..

[16] Sasaki K., Ose T., Okamoto N., Maenaka K., Tanaka T., Masai H., Saito M., Shirai T., Kohda D. (2007). Structural basis of the 3’-end recognition of a leading strand in stalled replication forks by PriA. EMBO J..

[17] Szymanski M.R., Jezewska M.J., Bujalowski W. (2011). Binding of two PriA-PriB complexes to the primosome assembly site initiates primosome formation. J. Mol. Biol..

[18] Cadman C.J., Lopper M., Moon P.B., Keck J.L., McGlynn P. (2005). PriB stimulates PriA helicase via an interaction with single-stranded DNA. J. Biol. Chem..

[19] Liu J., Nurse P., Marians K.J. (1996). The ordered assembly of the phiX174-type primosome. III. PriB facilitates complex formation between PriA and DnaT. J. Biol. Chem..

[20] Dong J., George N.P., Duckett K.L., DeBeer M.A., Lopper M.E. (2010). The crystal structure of Neisseria gonorrhoeae PriB reveals mechanistic differences among bacterial DNA replication restart pathways. Nucleic Acids Res..

[21] Huang C.Y., Hsu C.H., Sun Y.J., Wu H.N., Hsiao C.D. (2006). Complexed crystal structure of replication restart primosome protein PriB reveals a novel single-stranded DNA-binding mode. Nucleic Acids Res..

[22] Lopper M., Holton J.M., Keck J.L. (2004). Crystal structure of PriB, a component of the Escherichia coli replication restart primosome. Structure.

[23] Liu J.H., Chang T.W., Huang C.Y., Chen S.U., Wu H.N., Chang M.C., Hsiao C.D. (2004). Crystal structure of PriB, a primosomal DNA replication protein of Escherichia coli. J. Biol. Chem..

[24] Masai H., Arai K. (1989). Escherichia coli dnaT gene function is required for pBR322 plasmid replication but not for R1 plasmid replication. J. Bacteriol..

[25] McCool J.D., Ford C.C., Sandler S.J. (2004). A dnaT mutant with phenotypes similar to those of a priA2::kan mutant in Escherichia coli K-12. Genetics.

[26] Black S.L., Dawson A., Ward F.B., Allen R.J. (2013). Genes required for growth at high hydrostatic pressure in Escherichia coli K-12 identified by genome-wide screening. PLoS One.

[27] Huang Y.H., Huang C.Y. (2013). The N-terminal domain of DnaT, a primosomal DNA replication protein, is crucial for PriB binding and self-trimerization. Biochem. Biophys. Res. Commun..

[28] Huang Y.H., Lin M.J., Huang C.Y. (2013). DnaT is a single-stranded DNA binding protein. Genes Cells.

[29] Szymanski M.R., Jezewska M.J., Bujalowski W. (2013). Energetics of the Escherichia coli DnaT protein trimerization reaction. Biochemistry.

[30] Szymanski M.R., Jezewska M.J., Bujalowski W. (2013). The Escherichia coli primosomal DnaT protein exists in solution as a monomer-trimer equilibrium system. Biochemistry.

[31] Otwinowski Z., Minor W. (1997). Processing of X-ray diffraction data collected in oscillation mode. Macromol. Crystallogr., Pt A.

[32] Collaborative Coputational Protject Number, 4 (1994). The CCP4 suite: programs for protein crystallography. Acta Crystallogr. D Biol. Crystallogr..

[33] Adams P.D., Afonine P.V., Bunkoczi G., Chen V.B., Davis I.W., Echols N., Headd J.J., Hung L.W., Kapral G.J., Grosse-Kunstleve R.W. (2010). PHENIX: a comprehensive Python-based system for macromolecular structure solution. Acta Crystallogr. D Biol. Crystallogr..

[34] Emsley P., Cowtan K. (2004). Coot: model-building tools for molecular graphics. Acta Crystallogr. D Biol. Crystallogr..

[35] Murshudov G.N., Vagin A.A., Dodson E.J. (1997). Refinement of macromolecular structures by the maximum-likelihood method. Acta Crystallogr. D Biol. Crystallogr..

[36] Vagin A., Teplyakov A. (1997). MOLREP: an automated program for molecular replacement. J. Appl. Crystallogr..

[37] Davis I.W., Leaver-Fay A., Chen V.B., Block J.N., Kapral G.J., Wang X., Murray L.W., Arendall W.B., Snoeyink J., Richardson J.S. (2007). MolProbity: all-atom contacts and structure validation for proteins and nucleic acids. Nucleic Acids Res..

[38] Wiedemann C., Bellstedt P., Gorlach M. (2013). CAPITO–a web server-based analysis and plotting tool for circular dichroism data. Bioinformatics.

[39] Bocquet N., Bizard A.H., Abdulrahman W., Larsen N.B., Faty M., Cavadini S., Bunker R.D., Kowalczykowski S.C., Cejka P., Hickson I.D. (2014). Structural and mechanistic insight into Holliday-junction dissolution by topoisomerase IIIalpha and RMI1. Nat. Struct. Mol. Biol..

[40] Arai K., McMacken R., Yasuda S., Kornberg A. (1981). Purification and properties of Escherichia coli protein i, a prepriming protein in phi X174 DNA replication. J. Biol. Chem..

[41] Shamoo Y., Friedman A.M., Parsons M.R., Konigsberg W.H., Steitz T.A. (1995). Crystal structure of a replication fork single-stranded DNA binding protein (T4 gp32) complexed to DNA. Nature.

[42] Brenner S.L., Zlotnick A., Griffith J.D. (1988). RecA protein self-assembly. Multiple discrete aggregation states. J. Mol. Biol..

[43] Dickey T.H., Altschuler S.E., Wuttke D.S. (2013). Single-stranded DNA-binding proteins: multiple domains for multiple functions. Structure.

[44] Wang W., Ding J., Zhang Y., Hu Y., Wang D.C. (2014). Structural insights into the unique single-stranded DNA-binding mode of Helicobacter pylori DprA. Nucleic Acids Res..

[45] Lunde B.M., Moore C., Varani G. (2007). RNA-binding proteins: modular design for efficient function. Nat. Rev. Mol. Cell Biol..

[46] Duderstadt K.E., Chuang K., Berger J.M. (2011). DNA stretching by bacterial initiators promotes replication origin opening. Nature.

[47] Chen Z., Yang H., Pavletich N.P. (2008). Mechanism of homologous recombination from the RecA-ssDNA/dsDNA structures. Nature.

