# Supplementary material for: Crystal structure of DnaT84–153-dT10 ssDNA complex reveals a novel single-stranded DNA binding mode
Source: Nucleic Acids Res. 2014 Jul 22;42(14):9470–83. doi: 10.1093/nar/gku633 (PMC4132743; doi:10.1093/nar/gku633)
Supplement: SUPPLEMENTARY DATA [file supp_42_14_9470__index.html]

Crystal structure of DnaT84–153-dT10 ssDNA complex reveals a novel single-stranded DNA binding mode — SUPPLEMENTARY DATA 

# Crystal structure of DnaT84–153-dT10 ssDNA complex reveals a novel single-stranded DNA binding mode

## SUPPLEMENTARY DATA

**Files in this Data Supplement:**

- SUPPLEMENTARY DATA
