# Supplementary material for: Crystal structure of DnaT84–153-dT10 ssDNA complex reveals a novel single-stranded DNA binding mode
Source: Nucleic Acids Res. 2014 Jul 22;42(14):9470–83. doi: 10.1093/nar/gku633 (PMC4132743; doi:10.1093/nar/gku633)
Supplement: SUPPLEMENTARY DATA [file supp_gku633_nar-01027-z-2014-File011.pdf]

Table S1. DNA substrates used for studies.

**Name**	**Sequence**	**Study**
dT10	5’ - TTTTTTTTTT - 3’	EMSA
dT15	5’ - TTTTTTTTTTTTTTT -3’	X-ray
dT30	5’ - TTTTTTTTTTTTTTTTTTTTTTTTTTTTTT -3’	EMSA
5F-dT15	5’ - FAM - TTTTTTTTTTTTTTT -3’	FPA
5F-dA15	5’ - FAM - AAAAAAAAAAAAAAA -3’	FPA
5F-dT30	5’ - FAM - TTTTTTTTTTTTTTTTTTTTTTTTTTTTTT -3’	FPA
5F-dA30	5’ - FAM - AAAAAAAAAAAAAAAAAAAAAAAAAAAAAA -3’	FPA
5F-dC30	5’ - FAM - CCCCCCCCCCCCCCCCCCCCCCCCCCCCCC - 3’	FPA
5F-d(CA)$_{15}$	5’ - FAM - CACACACACACACACACACACACACACACA -3’	FPA
5F-d(TG)$_{15}$	5’ - FAM - TGTGTGTGTGTGTGTGTGTGTGTGTGTGTG -3’	FPA
5F-d(TGG)$_{10}$	5’ - FAM - TGGTGGTGGTGGTGGTGGTGGTGGTGGTGG -3’	FPA
5F-d(TGGG)$_{7}$	5’ - FAM - TGGGTGGGTGGGTGGGTGGGTGGGTGGG -3’	FPA

Table S2 Data collection and refinement statistics.

	$DnaT^{84\text{-}153}$-dT10[a]
**Data collection**	
Wavelength(Å)	0.97930
Space group	P 1
Cell dimensions	
a, *b*, *c* (Å)	47.14, 47.42, 54.14
α, *β*, *γ* (°)	88.34, 86.25, 71.24
Resolution (Å)	27.54 - 1.96 (2.03 -1.96)[b]
Unique reflections	30764 (2970)
Rmerge (%)	0.092(0.301)
I/σI	14.7(7.7)
Completeness (%)	97.1(96.6)
Redundancy	3.8
**Refinement**	
Rwork / Rfree	0.189 /0.229
Total No. atoms	3372
Residues	359
dT10	1
Waters	257
Mean B-factors	42.75
**R.m.s deviations**	
Bond lengths (Å)	0.0093
Bond angles (°)	1.2086
Most favored	99.4
Additional allowed	0.6
Disallowed regions	0

[a] dT10 is short for the dT10 ssDNA.

[b] Values in parentheses are for highest-resolution shell.

Table S3. Structural comparisons of DnaT with structurally similar proteins using DALI.

Protein	PDB ID	*Z*-score	RMSD	Sequence identity (%)	Number of Cα atoms superimposed
RMI1	4CHT	4.3	3.0	7	58
RMI1	4CGY	3.7	3.0	10	58
Probable RNA polymerase sigma-C factor	2O7G	3.5	3.1	13	64
Retinoblastoma-associated protein	4ELJ	3.4	3.0	8	49
Amylosucrase	1MW0	3.3	3.6	9	56
Amylosucrase	IMW1	3.3	3.6	9	56
Neurocalcin-delta	1BJF	3.2	3.4	10	50
RNA polymerase sigma factor	1H3L	3.2	3.1	11	56
RNA polymerase sigma factor	4LUP	3.1	3.3	8	63
ATP-dependent protease Lon	3KLJ	3.1	3.3	10	62
Rap1A protein	3ZFI	3.0	3.2	2	63
Rap1A	4BI8	2.9	3.2	2	63
Uncharacterized protein YmgD	2LRM	2.9	3.0	10	60
Protein SifA	3HW2	2.9	3.0	6	51
RNA polymerase sigma factor	2MAP	2.9	3.3	8	63
Phosphoribosylformylglycinamidine	4LGY	2.9	2.8	11	45
RNA polymerase sigma-E factor	1OR7	2.9	3.4	8	64
Uncharacterized protein YmgD	2LRY	2.9	3.0	8	62
L-seryl-tRNA(SEC) selenium transferase	3WLH	2.9	2.9	10	50
L-seryl-tRNA(SEC) selenium transferase	3WLK	2.9	2.9	10	51

SUPPLEMENTARY MATERIALS.

Figure S1

Four-step processes of DnaT crystals. (A) Crystals first appeared as fine needles in 3-4 days; these needles were too small to diffract well. (B) Some of the crystals became small sticks, which were large enough to diffract well for data collection after 1-2 weeks. (C) Crystals cracked after 2-3 weeks. (D) All of the crystals disappeared after 3-4 weeks.

Figure S2

SDS-PAGE and mass spectra assays of the different samples. (A) Coomassie Blue-stained 12% SDS-PAGE of the purified DnaT. (B) Coomassie Blue-stained 16% SDS-PAGE of the precipitate droplets and crystal droplets. The stars represent the bands that were used for the mass spectra assays. (C) Mass spectra results of the gels for Band1, Band2, and Band3. The mass spectra results of two bands from the precipitate droplets were the same as the crystal droplets (data not shown).

Figure S3

The 2Fo-Fc electron density of dT10 ssDNA contoured at 1.0-σ. (A) The 2Fo-Fc electron density of ssDNA in the 1.96 Å DnaT$^{84\text{-}153}$–dT10 complex structure. (B) The 2Fo-Fc electron density of ssDNA in the 2.83 Å DnaT$^{84\text{-}153}$–dT10 complex structure.

Figure S4

Structure superimpositions between the the N terminal of RMI1 and DnaT$^{84\text{-}153}$. The N terminal of RMI1 (from Asn^{2} to Arg^{62}) is shown in light blue, while DnaT$^{84\text{-}153}$ is shown in yellow.

Figure S5

Structural comparisons and EMSA assays. (A) EMSA result of dT10 ssDNA to full length DnaT. The stars represent the band position of the DnaT-dT10 ssDNA complex. (B) FPAs of DnaT with different types of 5´-FAM-labelled ssDNA substrates. The data of dA15 and dT15 were fitted according to the equation (see Methods). Error bars represent the standard deviation of three independent measurements. (C) Size exclusion chromatography analysis of the DnaT and its mutants. The assembly of DnaT and site-directed mutant Y127A, W128A, Y127A/W128A, K133A, F135A, K143A, and R146A proteins were determined using a Superdex 200 column (10/300 GL) (GE Healthcare). The dashed lines indicate the elution positions of the standard proteins. (D) Gel-filtration analysis of DnaT$^{84\text{-}179}$ and DnaT$^{84\text{-}153}$ performed on a Superdex 75 column (10/300 GL) (GE Healthcare). The theoretical molecular weights of the DnaT$^{84\text{-}179}$ dimer and DnaT$^{84\text{-}153}$ dimer in solution (buffer A) are 20 kDa and 14 kDa. The column was calibrated with proteins of known molecular masses (see Methods).

Figure S6

Negative staining EM results of different samples. (A) EM result of phiX174 ssDNA. (B-C) EM results of DnaT or DNase I pretreated DnaT. (D) EM result of DnaT-dT50 ssDNA complex. According to the proposed model in the Fig 6B-D, the length of DnaT bound dT50 ssDNA is about 30nm. The length of the particle could be estimated based on the green and red ruler. (E-F) DnaT-phiX174 ssDNA filaments observed by negative staining EM.

Figure S7

Negative staining EM results and two models. (A-B) EM results of dT50 ssDNA control and DnaT$^{84\text{-}179}$ control. (C) No visible filament of DnaT$^{84\text{-}179}$-phiX174 ssDNA filament could be observed by negative staining EM in the experimental condition. (D) The model illustrates dimeric DnaT$^{84\text{-}179}$ bind to ssDNA randomly. The elements used in the model are similar to Fig 6D. (E) Oligomerized DnaTs bind to ssDNA with higher cooperative effect to form a filament.

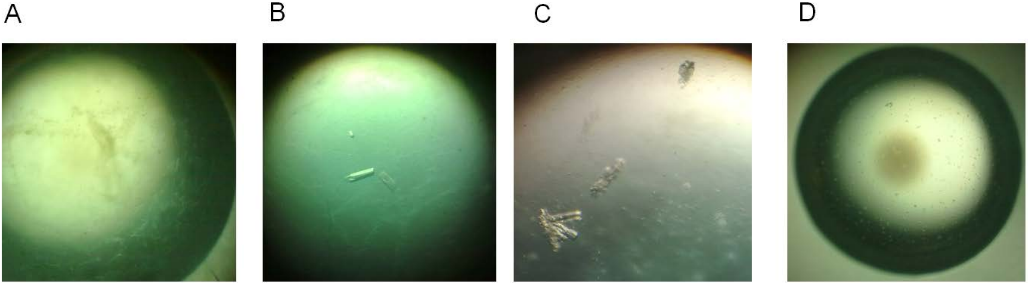
A
B
C
D

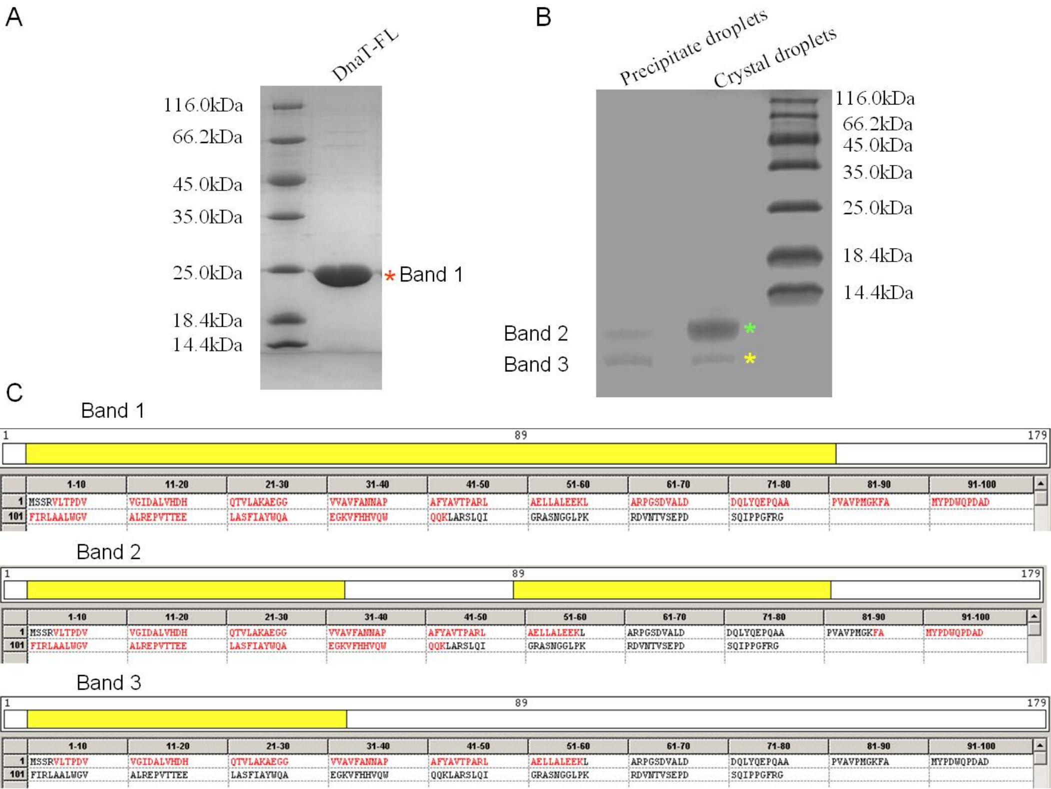

A
DnaT-FL
116.0kDa
66.2kDa
45.0kDa
35.0kDa
25.0kDa
18.4kDa
14.4kDa
Band 1
B
Precipitate droplets
Crystal droplets
116.0kDa
66.2kDa
45.0kDa
35.0kDa
25.0kDa
18.4kDa
14.4kDa
Band 2
Band 3
C
Band 1
1
89
179
1-10 11-20 21-30 31-40 41-50 51-60 61-70 71-80 81-90 91-100
1 MSSRVLTPDV VGIDALVHDH QTVLAKAEGG VVAVFANNAP AFYAVTPARL AELLALEEKL ARPGSDVALD DQLYQEPQAA PVAVPMGKFA MYPDWQPDAD
101 FIRLAALWGV ALREPVTTEE LASFIAYWQA EGKVFHHVQW QQKLARSLQI GRASNGGLPK RDVNTVSEPD SQIPPGFRG
Band 2
1
89
179
1-10 11-20 21-30 31-40 41-50 51-60 61-70 71-80 81-90 91-100
1 MSSRVLTPDV VGIDALVHDH QTVLAKAEGG VVAVFANNAP AFYAVTPARL AELLALEEKL ARPGSDVALD DQLYQEPQAA PVAVPMGKFA MYPDWQPDAD
101 FIRLAALWGV ALREPVTTEE LASFIAYWQA EGKVFHHVQW QQKLARSLQI GRASNGGLPK RDVNTVSEPD SQIPPGFRG
Band 3
1
89
179
1-10 11-20 21-30 31-40 41-50 51-60 61-70 71-80 81-90 91-100
1 MSSRVLTPDV VGIDALVHDH QTVLAKAEGG VVAVFANNAP AFYAVTPARL AELLALEEKL ARPGSDVALD DQLYQEPQAA PVAVPMGKFA MYPDWQPDAD
101 FIRLAALWGV ALREPVTTEE LASFIAYWQA EGKVFHHVQW QQKLARSLQI GRASNGGLPK RDVNTVSEPD SQIPPGFRG

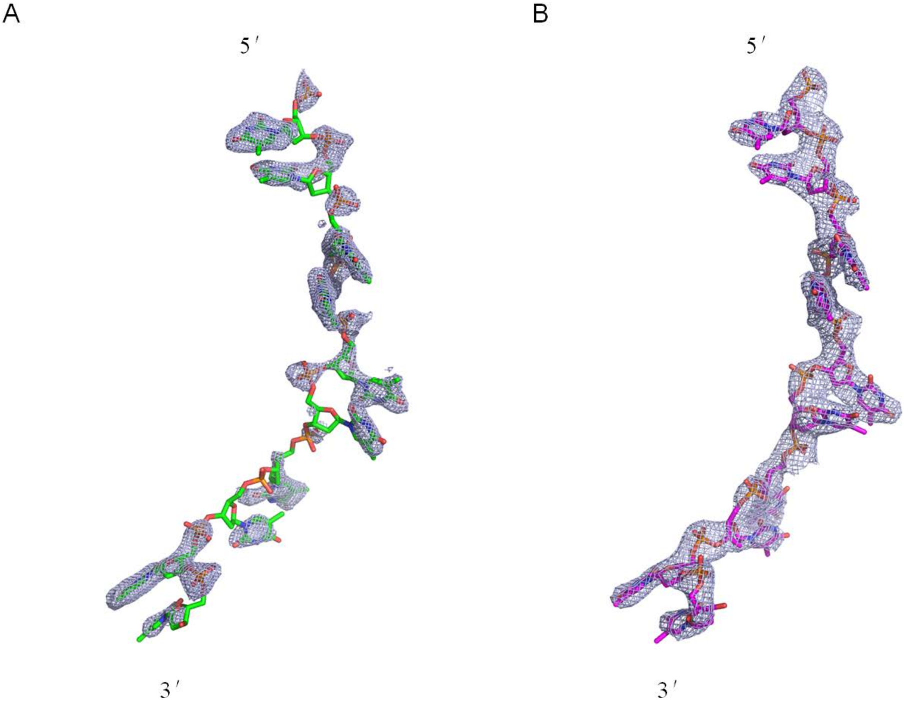
A
5′
3′
B
5′
3′

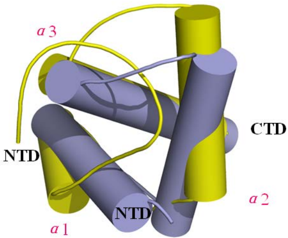
α3
NTD
CTD
NTD
α1
α2

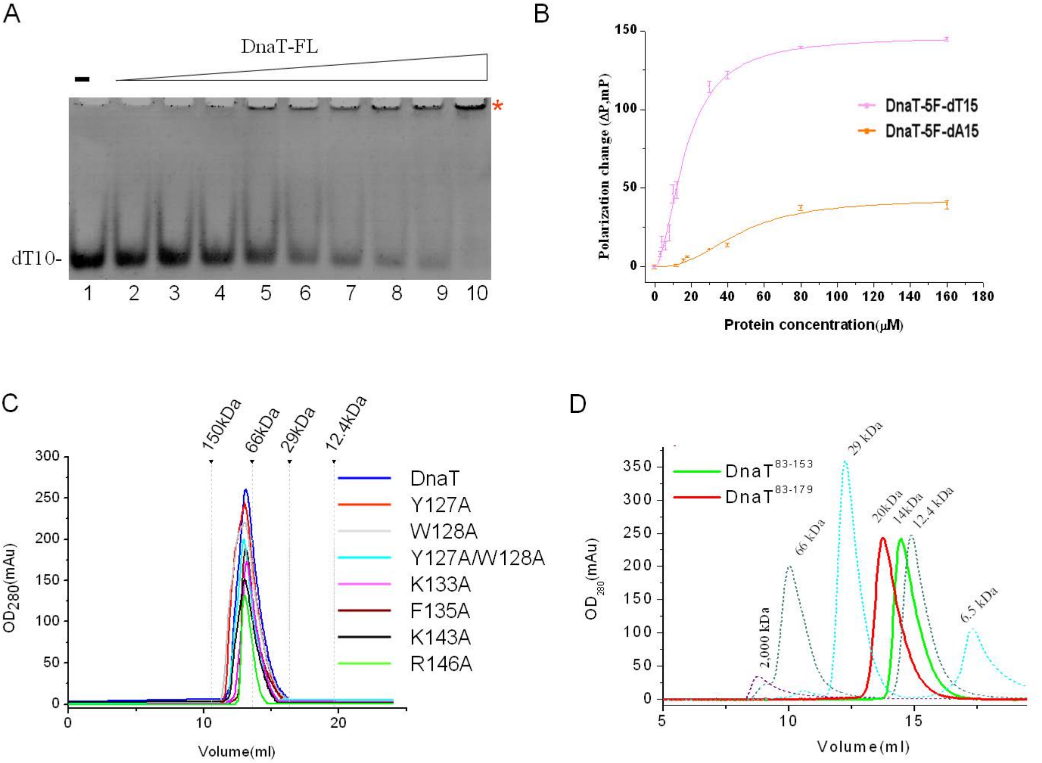
A
DnaT-FL
dT10-
1 2 3 4 5 6 7 8 9 10
B
Polarization change (ΔP,mP)
DnaT-5F-dT15
DnaT-5F-dA15
Protein concentration(μM)
C
150kDa
66kDa
29kDa
12.4kDa
DnaT
Y127A
W128A
Y127A/W128A
K133A
F135A
K143A
R146A
OD280(mAu)
Volume(ml)
D
DnaT83-153
DnaT83-179
2,000 kDa
66 kDa
29 kDa
20kDa
14kDa
12.4 kDa
6.5 kDa
OD280(mAu)
Volume(ml)

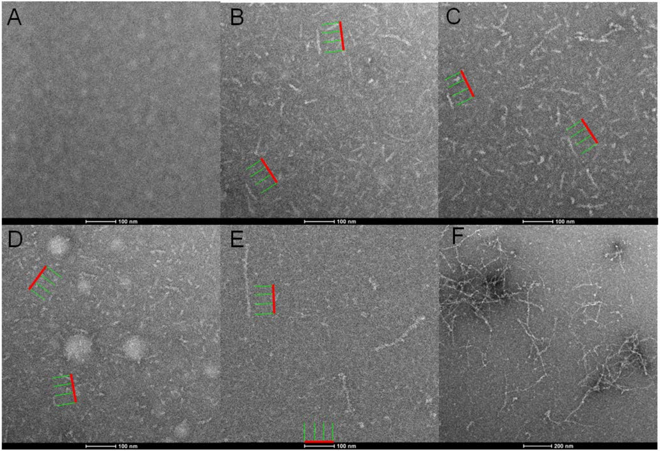
A
B
C
D
E
F
100 nm
100 nm
100 nm
100 nm
100 nm
200 nm

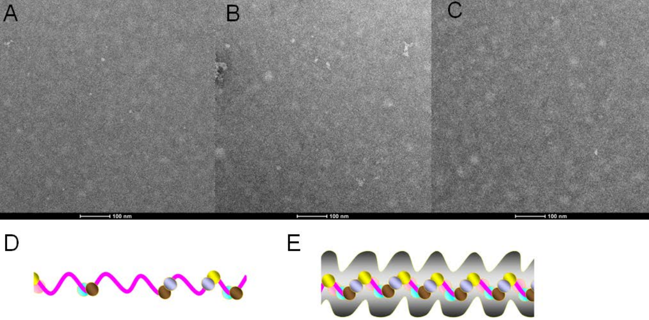
A
B
C
100 nm
100 nm
100 nm
D
E
